# RNF144A and RNF144B: Important molecules for health

**DOI:** 10.1515/biol-2025-1130

**Published:** 2025-08-01

**Authors:** Wang Jiang, Yi Liang, Min Han, Wenhua He, Kun Chen, Chongtian Deng, Yueming Shen

**Affiliations:** Department of Digestive Diseases, The Affiliated Changsha Central Hospital, Hengyang Medical School, University of South China, 161 Shaoshan Road, Changsha, 410000, China; Department of Gastrointestinal Surgery, The Second Affiliated Hospital, Hengyang Medical School, University of South China, 35 Jiefang Road, Hengyang, 421000, China; Department of Cardiovascular Diseases, The Affiliated Changsha Central Hospital, Hengyang Medical School, University of South China, 161 Shaoshan Road, Changsha, 410000, China; Department of Gastroenterology, Jiangxi Provincial Key Laboratory of Digestive Diseases, Jiangxi Clinical Research Center for Gastroenterology, Digestive Disease Hospital, The First Affiliated Hospital, Jiangxi Medical College, Nanchang University, Nanchang, Jiangxi, China; Department of Orthopaedics, The Affiliated Changsha Central Hospital, Hengyang Medical School, University of South China, Changsha, Hunan, China; The First Clinical College, Changsha Medical University, 1501 Leifeng Avenue, Changsha, 411200, China

**Keywords:** RNF144, RNF144A, RNF144B, disease

## Abstract

RNF144 family proteins, including RNF144A and RNF144B, members of the RING-between-RING domain-containing ubiquitin E3 ligase family, serve as critical regulators of protein ubiquitination. Despite increasing research attention in recent years, particularly regarding their distinct functional roles in pathophysiological processes, a comprehensive synthesis of existing findings remains absent. To address this knowledge gap, we conducted a systematic literature search in PubMed using the following query terms: “RNF144,” “RNF144A,” “RNF144B,” “PIR2,” “IBRDC2,” and “P53RFP.” This review systematically examines current evidence regarding the molecular mechanisms and pathophysiological significance of RNF144A/B across various disease systems. Through critical analysis of structural characteristics, substrate interactions, and signaling pathways, we aim to clarify their dual roles in cellular homeostasis and disease pathogenesis. This synthesis not only consolidates current understanding but also identifies key knowledge gaps requiring further investigation, particularly regarding isoform-specific functions and therapeutic targeting potential.

## Graphical abstract

**Figure:**
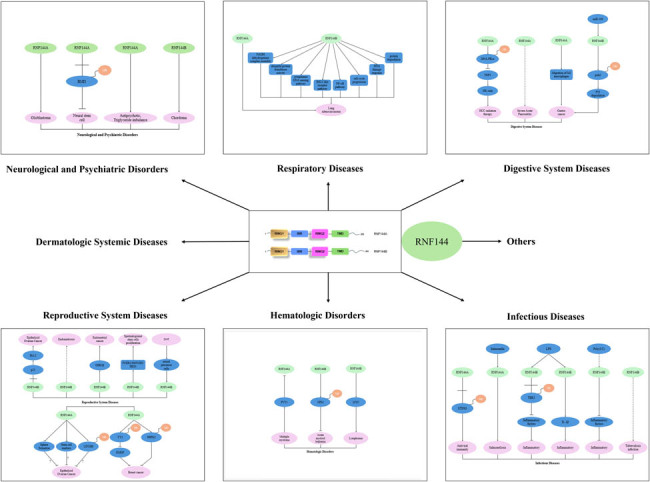

## 1 Introduction

Ubiquitination, a critical post-translational modification, regulates protein homeostasis through dynamic control of protein stability, subcellular localization, and functional activity [1]. Ubiquitination means the binding of ubiquitin molecules to target proteins via a cascade of enzymatic reactions induced by the ubiquitin-proteasome system [2,3]. Ubiquitination enzymes contain ubiquitin-activating enzymes (E1), ubiquitin-conjugating enzymes (E2), and ubiquitin-ligase enzymes (E3) [4]. E3 ligases are categorized into four mechanistically distinct classes based on their structural domains: Homologous to E6AP C-Terminus-type (HECT) containing a catalytic HECT domain; RING-type (Really Interesting New Gene) featuring RING finger domains mediating E2 interactions; RING-between-RING-type (RBR) employing a tripartite RING1-IBR-RING2 architecture; PCAF_N-type characterized by N-terminal p300/CBP-associated factor (PCAF) domains [5,6].

**Figure 1:**
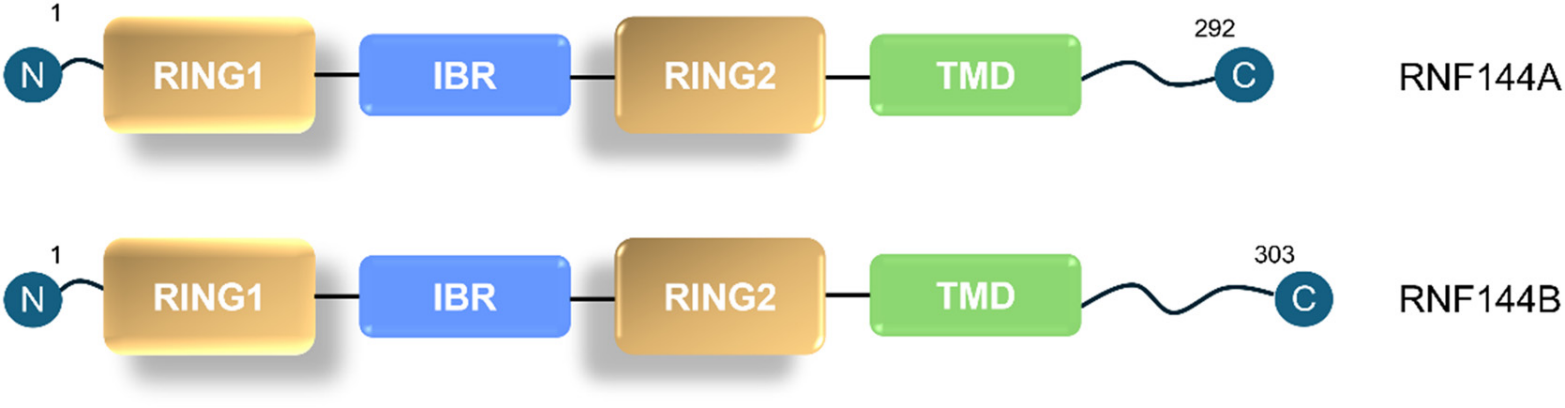
Schematic structure of RNF144A/RNF144B.

The RNF144 family proteins, comprising RNF144A and RNF144B (alternatively designated as PIR2, IBRDC2, and P53RFP), represent a distinct class of RBR E3 ubiquitin ligases. Structural analyses reveal a conserved bipartite architecture: an N-terminal RBR domain (RING1-IBR-RING2) and a C-terminal transmembrane (TM) domain (Figure 1) [7,8,9,10]. Mechanistically, the RBR domain facilitates specific E2 ubiquitin-conjugating enzyme recruitment [11], while the TM domain exhibits dual functionality in membrane association and allosteric regulation of ligase activity [7]. Notably, emerging evidence indicates intrinsic functional significance of the TM domain beyond its structural role [12]. Despite growing recognition of RNF144 family involvement in diverse pathological conditions, systematic analyses of their disease-associated mechanisms remain conspicuously absent in current literature. We conducted a literature search in PubMed using the following query terms: “RNF144,” “RNF144A,” “RNF144B,” “PIR2,” “IBRDC2,” and “P53RFP” and ranging from 1982 to 2024. The result of search demonstrates the literatures about RNF144 family proteins were concentrating on recently 10 years. This review comprehensively examines regulatory mechanisms and therapeutic implications of RNF144.

## 2 RNF144 family proteins with neurological and psychiatric diseases

**Figure 2:**
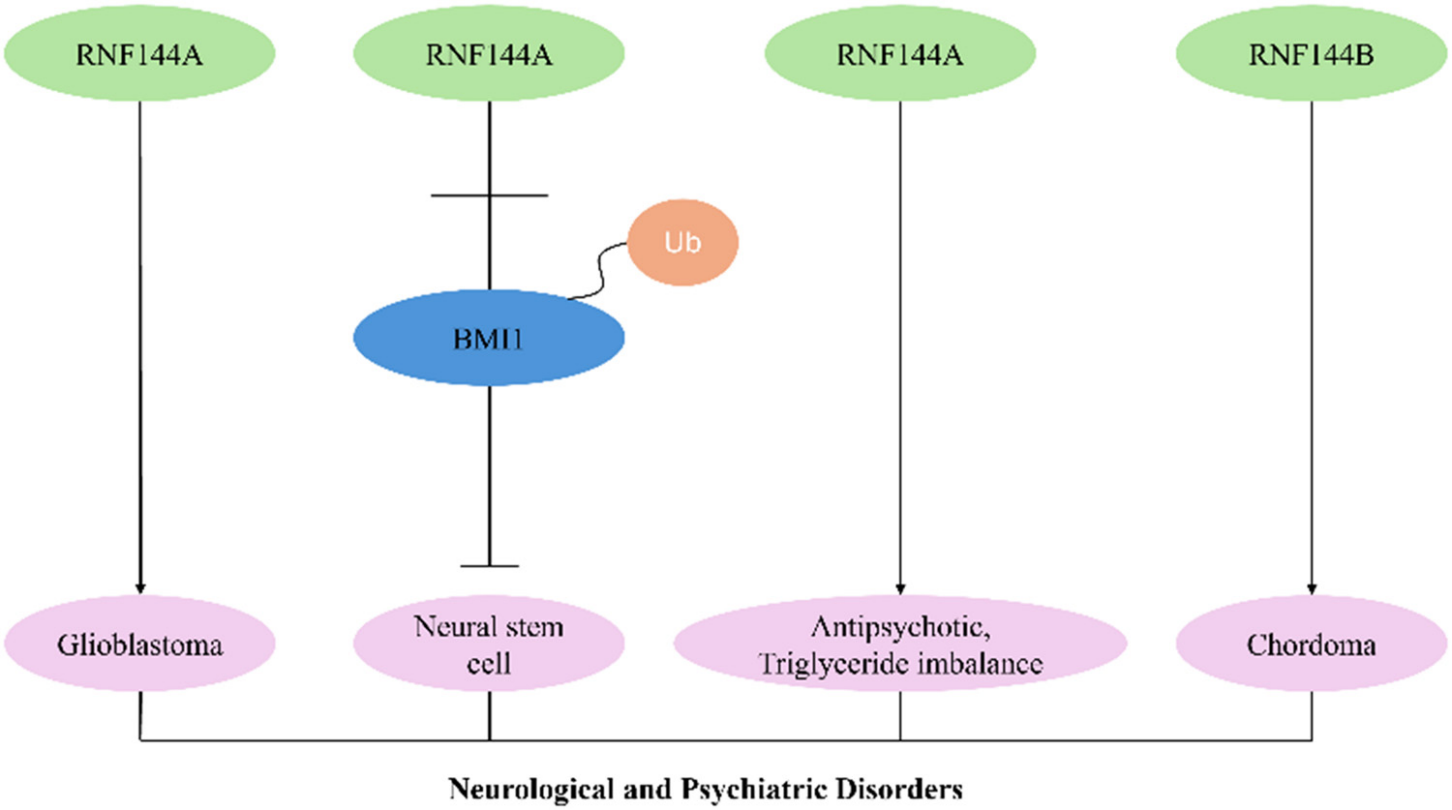
RNF144A/RNF144B and neurological and psychiatric disorders.

The RNF144 protein family demonstrates significant pathophysiological relevance across neurological and psychiatric disorders, including glioma pathogenesis, neural stem cell regulation, schizophrenia susceptibility, and chordoma development (Figure 2).

### 2.1 RNF144 family proteins with neurological diseases

The RNF144A expression is lower in glioblastoma than in low-grade gliomas and is negatively correlated with patient survival. The B cell-specific Moloney murine leukemia virus integration site 1 (BMI1), originally characterized as an oncoprotein, executes critical cellular functions through three primary mechanisms: (1) inhibition of premature senescence, (2) maintenance of stem cell self-renewal capacity, and (3) enhancement of cellular stress adaptation [13,14,15]. Within neural systems, BMI1 serves as a key regulator of proliferation in both neural progenitor cells and neural stem cell populations [16]. Notably, RNF144A exhibits a dual regulatory relationship with BMI1, demonstrating negative correlation with BMI1 activation status, and positive association with BMI1 inhibitory signaling pathways [15]. Mechanistic studies employing co-immunoprecipitation assays confirm direct RNF144A-BMI1 physical interaction, through which RNF144A catalyzes BMI1 ubiquitination and subsequent proteasomal degradation [15]. These findings imply RNF144A-mediated ubiquitin-dependent regulation of BMI1 as a potential modulator of neurodevelopmental processes. However, critical molecular details, including precise binding interfaces, ubiquitination acceptor lysine residues, and structural determinants of substrate recognition, remain to be fully elucidated.

The DHHC-family proteins demonstrate significant involvement in glioma pathogenesis [17]. Mechanistic studies reveal that ZDHHC18 and ZDHHC23 competitively link RNF144A to differentially modulate BMI1 polyubiquitination and proteostatic regulation [18]. Experimental manipulation of these DHHC isoforms demonstrates reciprocal functional relationships: ZDHHC18 overexpression attenuates ZDHHC23-RNF144A binding while elevating BMI1 protein levels, and ZDHHC23 overexpression disrupts ZDHHC18-RNF144A interaction while reducing BMI1 abundance [18].

RNF144B can promote chordoma progression [19,20]. Its expression was reported to be up-regulated in chordoma tissues, and knockdown of RNF144B led to inhibition of cell proliferation, migration, and invasion, whose specific mechanisms are to be further explored [20].

### 2.2 RNF144 family proteins with psychiatric diseases

A genome-wide association study (GWAS) conducted in a cohort of 738 schizophrenia patients receiving standard antipsychotic therapy identified RNF144A as a pharmacogenomic susceptibility locus associated with metabolic adverse effects. Functional annotations demonstrated this gene’s specific involvement in drug-induced triglyceride dysregulation, establishing it as a key mediator of antipsychotic-related metabolic disturbances [21]. Specific mechanisms by which RNF144A regulates schizophrenia are to be further explored.

## 3 RNF144 family proteins with respiratory diseases

**Figure 3:**
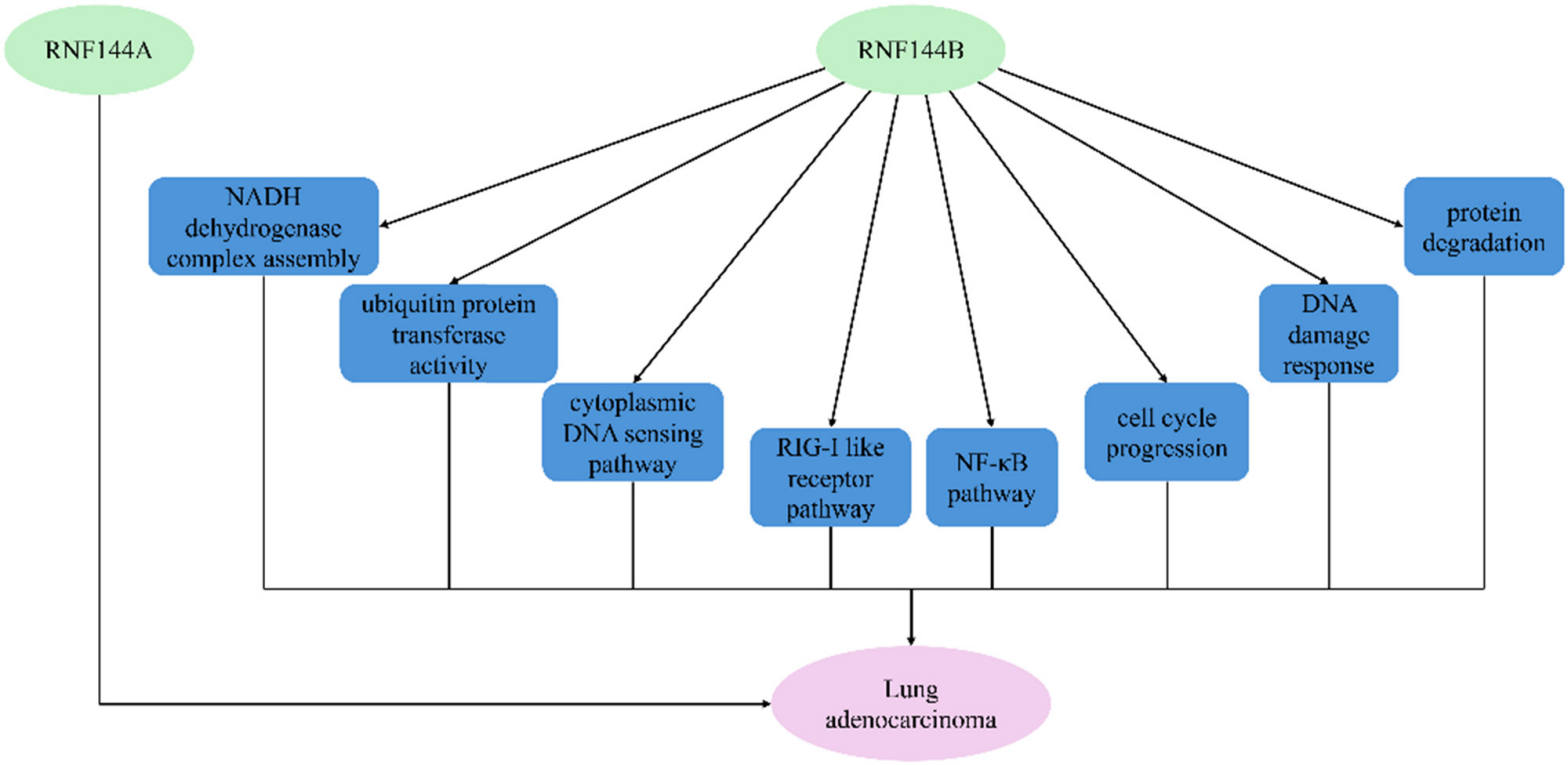
RNF144A/RNF144B and respiratory diseases.

RNF144 demonstrates predominant association with solid tumors of the respiratory system, exhibiting dual roles (Figure 3).

### 3.1 RNF144 family proteins promote respiratory diseases

A representative case involving a stage IVB lung adenocarcinoma (LUAD) patient harboring the ALK-RNF144A tyrosine kinase fusion demonstrated clinical significance. Following combination therapy with the third-generation ALK inhibitor lorlatinib, paclitaxel, and anlotinib, the patient achieved durable disease stabilization with progression-free survival exceeding 18 months [22]. There is a lack of research on this mechanism of action.

### 3.2 RNF144 families inhibit respiratory diseases

Integrated multi-omics analysis combining TCGA data mining, Kaplan-Meier survival modeling, and functional enrichment analyses (STRING, GO/KEGG, GSEA) systematically investigated RBR E3 ubiquitin ligases in LUAD pathogenesis [23]. The study identified RNF144B as a clinically relevant biomarker strongly correlated with advanced clinicopathological features and poor prognostic outcomes. Mechanistic multi-omics interrogation revealed RNF144B’s functional engagement in mitochondrial metabolic reprogramming (NADH dehydrogenase complex assembly), ubiquitination machinery (ubiquitin transferase activity), and immune regulation through cytoplasmic nucleic acid sensing (RIG-I-like receptor pathway) and inflammatory signaling (NF-κB pathway) [23]. Complementary functional studies demonstrated that the knockdown of RNF144B in human and murine LUAD models confers proliferative advantage and oncogenic transformation, mediated through cell cycle deregulation, defective DNA damage response, and proteostasis impairment [24]. Importantly, RNF144B deficiency induces chromosomal instability and mitotic catastrophe in LUAD cells, which have been associated with worse prognosis [24]. Unfortunately, none of these studies have elaborated on the pathways and target proteins through which RNF144B regulates LUAD.

## 4 RNF144 family proteins with digestive system diseases

**Figure 4:**
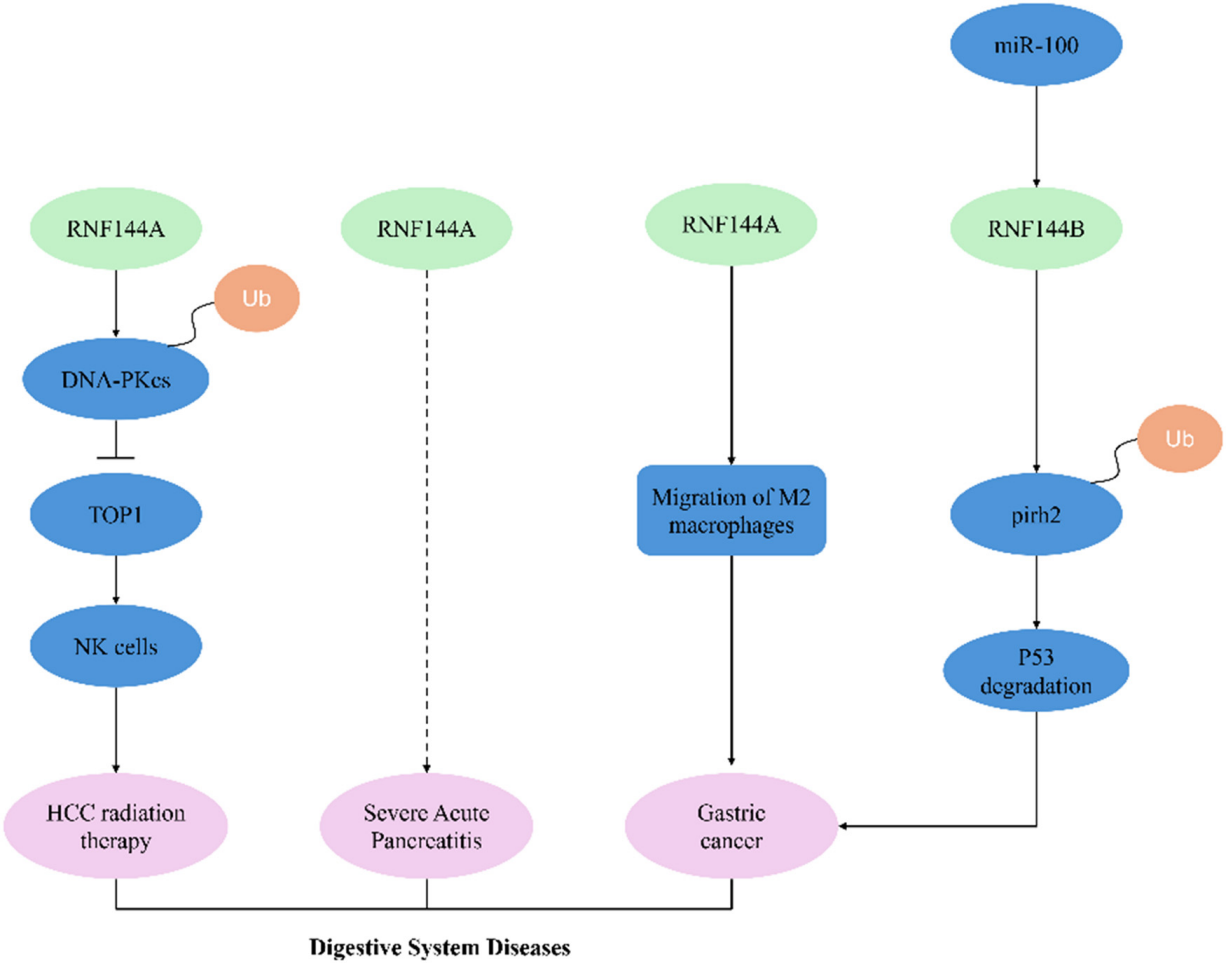
RNF144A/RNF144B and digestive system diseases.

The RNF144 protein family demonstrates significant pathological associations across digestive system malignancies, including hepatocellular carcinoma (HCC), gastric cancer, and severe acute pancreatitis (SAP) (Figure 4). Functional characterization reveals dual roles of RNF144 family proteins in gastrointestinal oncology.

### 4.1 RNF144 family proteins promote digestive system diseases

Integrative multi-platform analysis identifies RNF144A as a pathogenic driver in gastric adenocarcinoma, mechanistically linked to its capacity to orchestrate M2 macrophage polarization [25]. Notably, miR-100 overexpression in gastric cancer specimens establishes an oncogenic signaling axis through direct transcriptional activation of RNF144B. This miRNA-mediated regulation triggers ubiquitin-mediated proteasomal degradation of pirh2 (a p53-specific E3 ligase), consequently destabilizing tumor suppressor p53 and driving malignant progression [26].

Comprehensive dataset and bioinformatics analyses identified RNF144A as a dysregulated molecular signature along SAP pathophysiological continuum [27]. The expression of RNF144A was upregulated in SAP, highlighting the potential of using RNF144A as a predictor of SAP prognosis, which is closely associated with a variety of immune cells and may be a feature of regulating immune cell penetration in the microenvironment. This remains to be demonstrated by further *in vivo* and *in vitro* experiments. These findings suggest that RNF144A may be a potential target for developing SAP therapies [27].

### 4.2 RNF144 family proteins inhibit digestive system diseases

Single-cell transcriptomic profiling of CD133+ cancer stem cells identified RNF144A as a somatic mutation hotspot specifically enriched in hepatic metastatic lesions [28]. Mechanistic interrogation revealed a DNA repair regulatory axis involving this E3 ligase: RNF144A mediates ubiquitin-dependent degradation of DNA-PKcs through its canonical catalytic activity, thereby suppressing topoisomerase I (TOP1)-mediated DNA double-strand break repair [29]. This molecular cascade potentiates radiation-induced NK cell activation, ultimately conferring radiosensitization in HCC models [30].

## 5 RNF144 family proteins with reproductive system diseases

**Figure 5:**
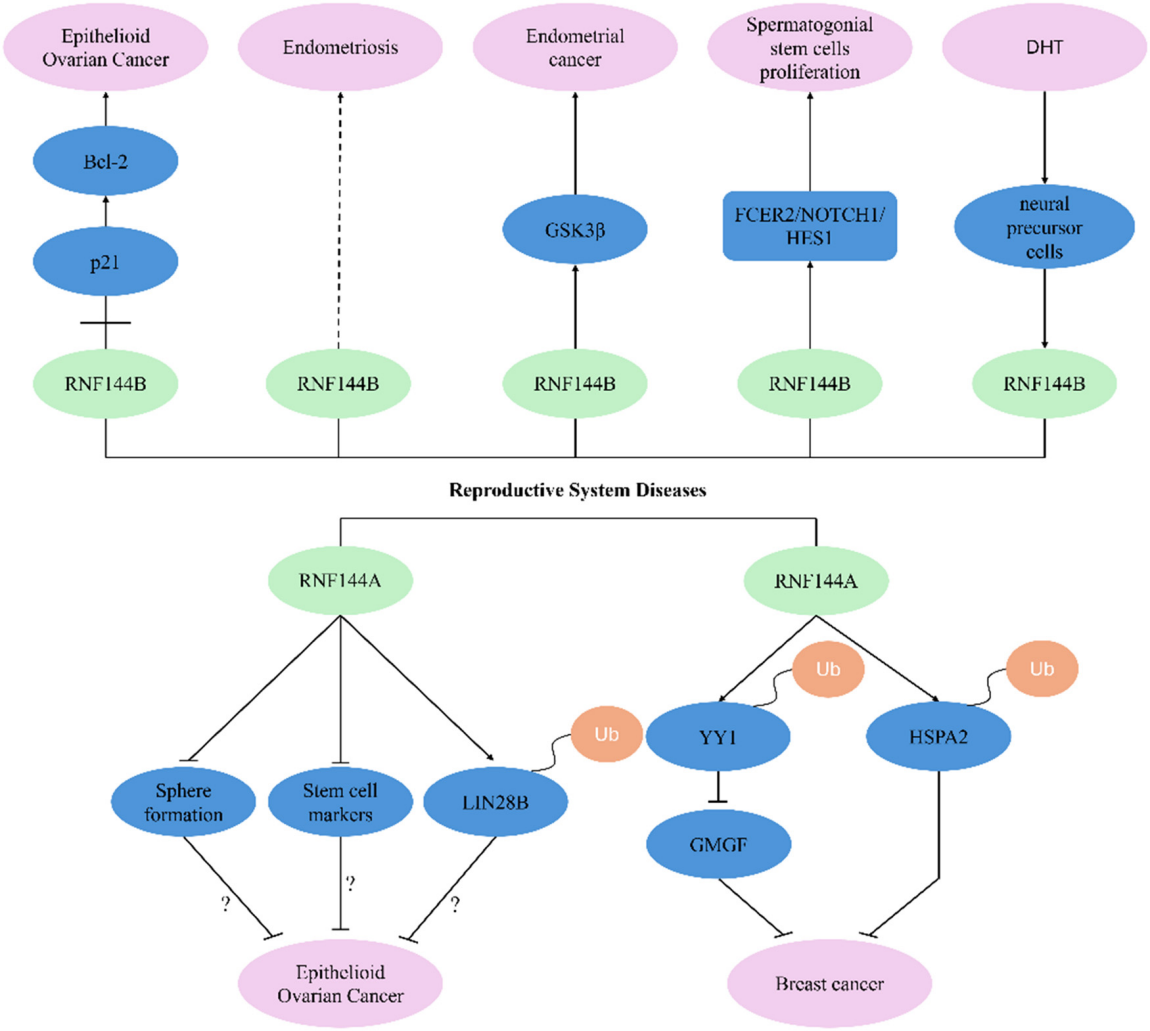
RNF144A/RNF144B and reproductive system diseases.

The RNF144 ubiquitin ligase family exhibits broad pathophysiological relevance across reproductive system disorders, spanning gynecological malignancies (epithelioid ovarian carcinoma, hormone receptor-positive breast cancer, endometrial adenocarcinoma) to benign conditions (endometriosis, spermatogonial stem cell dysregulation) (Figure 5). Functional characterization reveals dual roles of RNF144 family proteins in reproductive system diseases.

### 5.1 RNF144 family proteins promote reproductive system diseases

Through multi-dimensional bioinformatics interrogation integrating TCGA-OV, GSE140082, and GSE34526 datasets-encompassing differential transcriptomics, prognostic Cox modeling, pathway topology mapping, PPI network deconvolution, survival meta-analysis, and immunoblot validation, we identified RNF144B as a molecular nexus bridging polycystic ovary syndrome (PCOS) and ovarian carcinogenesis within a shared pathological continuum [31]. RNF144B plays an important role in the oncogenesis and metastatic dissemination of ovarian cancer, and the level of RNF144B is significantly higher in ovarian cancer tissues than in normal ovarian and benign ovarian tumor tissues. RNF144B interacts with p21 and regulates the degradation of the p21/p53 complex. RNF144B overexpression was reported to promote ovarian cancer growth and metastasis by inhibiting the expression of p53/p21/Bax and elevating the expression of Bcl-2 [32].

Integrative analysis of GWAS with cross-platform meta-analyses identified RNF144B as a novel susceptibility locus for endometriosis, though its precise molecular pathophysiology requires functional validation [33]. RNF144B is a potential target biomarker for endometrial cancer. Intriguingly, this E3 ligase exhibits cancer-specific proteomic dysregulation – while maintaining comparable mRNA levels in normal and malignant endometrium, RNF144B protein expression is exclusively detected in endometrial carcinoma specimens [34]. Mechanistically, RNF144B can stabilize the phosphorylation level of GSK3β downstream signaling molecules, and knockdown of the RNF144B gene in endometrial cancer cells inactivated GSK3β, leading to inhibition of cell proliferation [34].

It was shown that RNF144B promotes the proliferation and inhibits the apoptosis of human spermatogonial stem cells through the FCER2/NOTCH1/HES1 pathway. RNF144B interacts with FCER2, which downregulates the expression of the NOTCH2 structural domain N2ICD, while knockdown of human spermatogonial stem cells by RNF144B was revealed to decrease the FCER2, NOTCH2, and HES1 levels [35]. Moreover, RNF144B has been associated with androgen production. In an earlier study, RNA sequencing was used to detect the genes with altered expression in human neural precursor cells induced by dihydroxytestosterone, and it was found that the expression of RNF144B was significantly elevated [36].

### 5.2 RNF144 family proteins inhibit reproductive system diseases

Immunohistochemistry analyses reveal significant RNF144A downregulation in epithelioid ovarian cancer (EOC) specimens compared to benign controls [37]. Clinically, EOC patients with RNF144A-low tumors exhibit reduced overall survival [35]. The knockdown of RNF144A was reported to significantly enhance sphere formation and upregulate the stem cell markers in EOC cells, whereas RNF144A overexpression had the opposite effects [37]. LIN28B is an RNA-binding protein responsible for the post-transcriptional regulation of the let-7 family of microRNAs in invertebrates and mammals [38] and is highly expressed in progenitor and stem cells [39]. RNF144A was reported to induce the ubiquitinated degradation of LIN28B in EOC cells [37]. It is hypothesized that RNF144A promotes the degradation of LIN28B in EOC, thereby inhibiting the development of EOC, whose authenticity needs to be further investigated.

RNF144A inhibits breast cancer in three ways. Glia maturation factor-γ (GMFG) is a protein that regulates tumor progression, immune response modulation, and the tissue-specific tumor microenvironment [40]. It has been implicated in colorectal cancer, epithelial ovarian cancer, glioblastoma, low-grade glioma, squamous lung cancer, and ocular melanoma [40,41,42]. The transcription of GMFG was regulated by the transcription factor YY1. It was found that RNF144A can interact with YY1 to promote the ubiquitination-dependent degradation of YYI, thus inhibiting the GMFG expression. This mechanism explains how RNF144A inhibits the proliferation, migration, and invasion of breast cancer cells [43].

The RNF144A expression was downregulated in a group of primary breast cancers, and RNF144A overexpression in breast cancer cells inhibited proliferation, colony formation, migration, *in vitro* invasion, *in vivo* tumor growth, and metastasis. On the other hand, the knockdown of RNF144A promoted the malignant phenotype of breast cancer cells [44].

It was found that RNF144A interacts with the heat-shock protein family A member 2 (HSPA2), an oncoprotein, and promotes its ubiquitination degradation, thus negatively regulating breast cancer [44]. Methylation of the promoter of RNF144A can silence the RNF144A gene. There is a CpG island in the RNF144A promoter, and knockdown of the endogenous methyl CpG island-binding structural domain 4 (MBD4) of RNF144A in breast cancer upregulated the expression of RNF144A. This suggests that RNF144A is regulated by promoter methylation [45].

## 6 RNF144 family proteins with hematologic diseases

**Figure 6:**
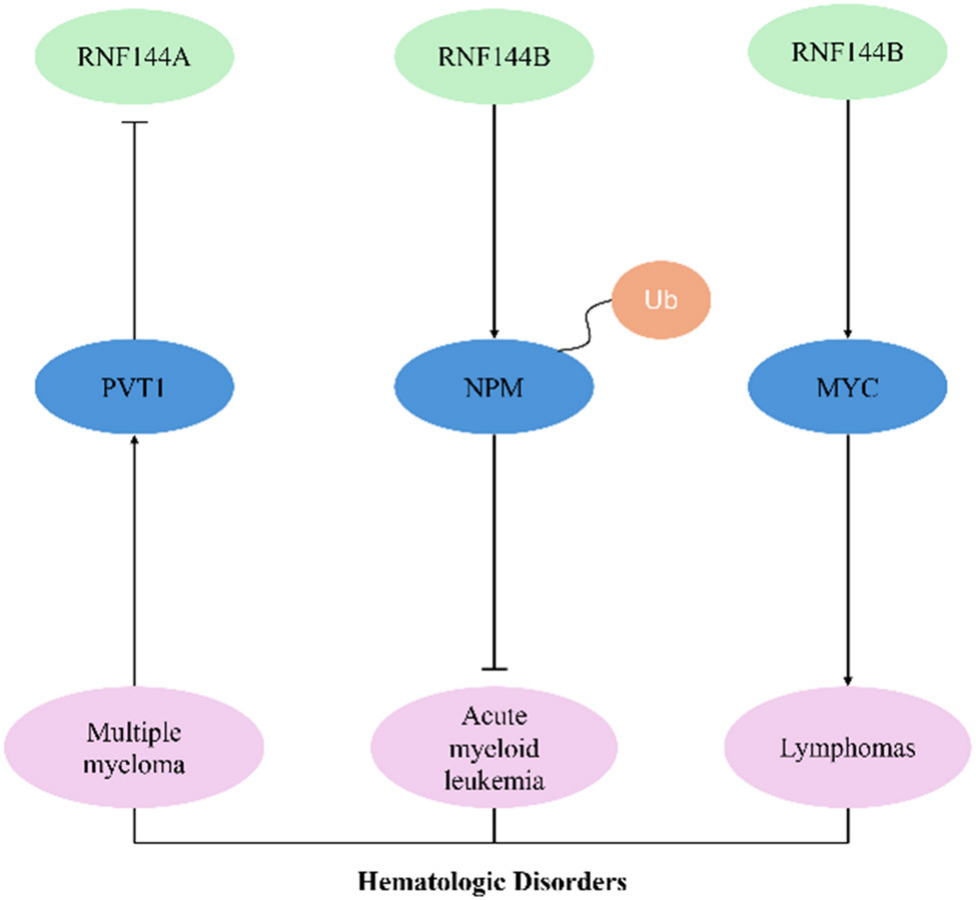
RNF144A/RNF144B and hematologic disorders.

RNF144 is associated with multiple myeloma, acute myeloid leukemia, and lymphomas (Figure 6). Plasmacytoma variant translocation 1 (PVT1), an interacting partner of tumor suppressor and pro-apoptotic gene silencing in multiple myeloma, was overexpressed in patients with poor prognosis, and inhibition of the PVT1 expression was found to promote the expression of RNF144A, which may be a new research direction in multiple myeloma [46].

RNF144B has been associated with leukemia. Nucleophosmin (NPM) is a multifunctional oligomeric protein involved in liquid-liquid phase separation, ribosome generation, histone chaperones, and transcriptional regulation [47,48]. NPM was downregulated in acute myeloid leukemia myeloid cells [49]. The knockdown of RNF144B significantly lowered the ubiquitination level of NPM and restored the stability of NPM, while RNF144B overexpression promoted the ubiquitination degradation of NPM [49]. In addition, it was found that knockdown of RNF144B, similar to knockdown of p53, accelerated the development of MYC-driven lymphomas, and the mechanism might be associated with cell cycle progression [50].

## 7 RNF144 family proteins with infectious diseases

**Figure 7:**
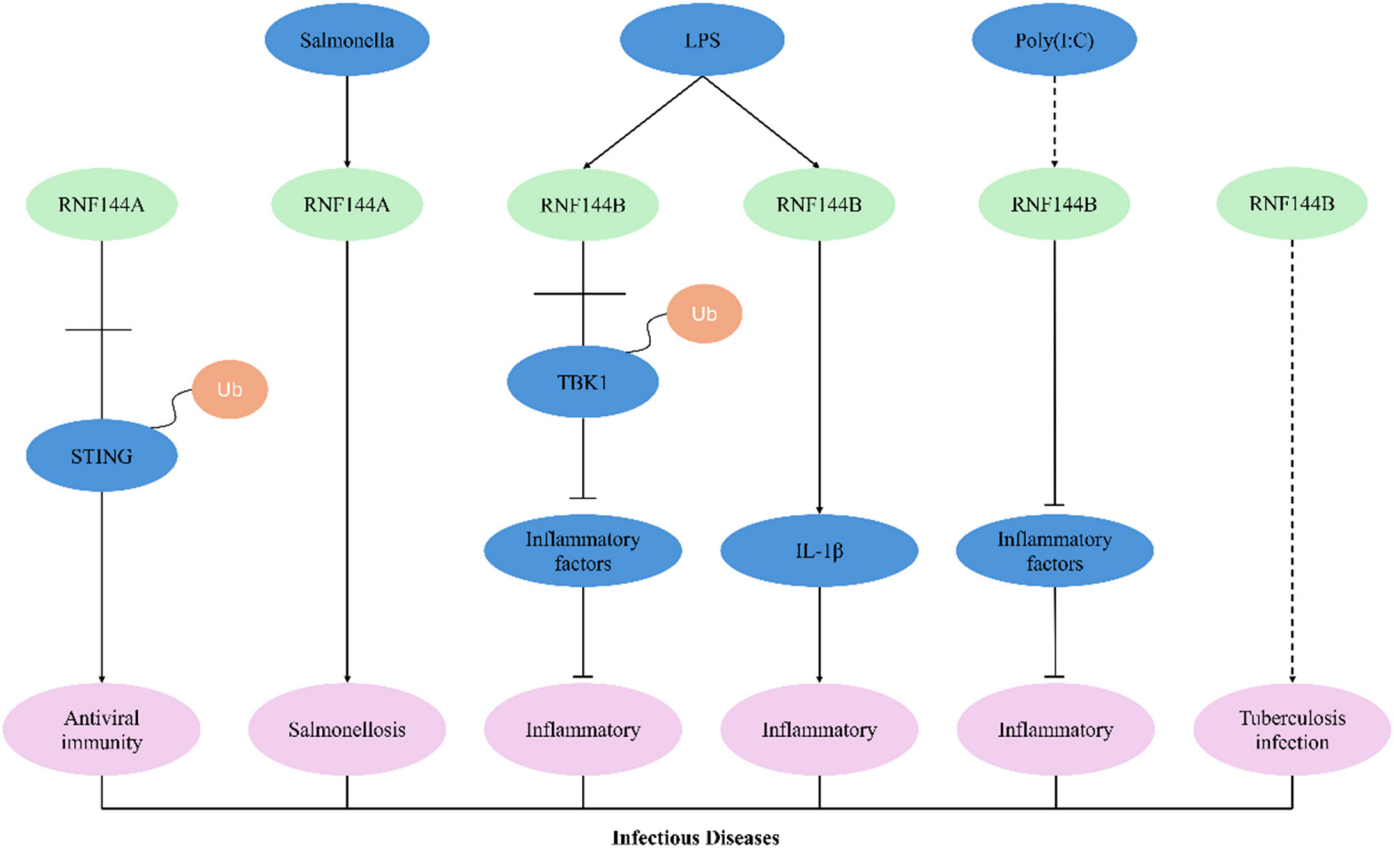
RNF144A/RNF144B and infectious diseases.

RNF144 is associated with antiviral immunity, salmonellosis, lipopolysaccharide (LPS)-induced inflammation, poly(I:C)-induced inflammation, and tuberculosis infection (Figure 7). STING is an interferon-stimulating factor that plays an important role in antiviral immunity. It was found that RNF144A can interact with STING and promote the ubiquitination of its K6 linkage at K236, thereby inducing downstream signaling molecules [51]. RNF144A overexpression was reported to upregulate HSV-1 or cytoplasmic DNA-induced immune responses, while RNF144A knockdown had the opposite effects. RNA144A exhibited a protective effect on the DNA virus-induced viral immunity in mice [51]. By infecting Atlantic salmon with Salmonella, it was found that RNF144A can be strongly induced by Salmonella through the mechanism correlated with the correlation of T cell function as detected by transcriptomics and quantitative PCR [52].

The expression level of RNF144B was significantly elevated in the peripheral blood mononuclear cells from patients with sepsis, as well as after induction of bone marrow stromal stem cells by LPS. Knockdown of RNF144B in BMDM cells resulted in increased release of inflammatory factors, suggesting that RNF144B can attenuate the inflammatory response caused by sepsis [53]. Mechanistically, RNF144B interacts with TNAK-binding kinase 1 (TBK1), and RNF144B deficiency can lead to impaired TBK1 activation but enhanced NF-κB activation and increased inflammatory factor release [53].

Another study found that RNF144B can inhibit LPS-induced inflammatory response. By interacting with the scaffold/dimerization structural domain of TBK1 through the in-between- ring (IBR) structural domain, RNF144B can inhibit TBK1 phosphorylation and K63-linked ubiquitination, consequently leading to TBK1 inactivation, IRF3 dephosphorylation, and decreased INF-β release. It was reported that the knockdown of RNF144B increased LPS-induced inflammatory responses [54]. However, a study yielded different results: LPS induced the upregulation of RNF144B in human macrophages and human macrophage-like THP-1 cells, the downregulation of RNF144B in HMDM cells, and a decrease in LPS-induced interleukin-1β (IL-1β) release, suggesting that RNF144B facilitates the LPS-induced IL-1β expression [55].

It was found that knockdown of RNF144B resulted in the upregulation of poly(I:C)-induced expression of the transcription factor IRF3 as well as inflammatory factors such as tumor necrosis factor α (TNFα), IL-6, and interferon I (IFN-I). This suggests that RNF144B is a negative regulator of poly(I:C)-induced inflammation [56]. RNF144B has been strongly associated with susceptibility to bovine tuberculosis, and genome-wide association analysis revealed that RNF144B is the gene most associated with single nucleotide polymorphisms on bovine autosomes [57,58].

## 8 RNF144 family proteins with dermatologic systemic diseases

RNF144B is an important influencer of keratinocyte proliferation and differentiation. RNF144B was found to bind and mediate the proteasomal degradation of ΔNp63α, and knockdown of RNF144B in primary keratinocytes resulted in increased accumulation of p21WAF1/CIP1 and P63 but severely impaired proliferation and differentiation of keratinocytes [59].

Dysregulation of the microRNA-31 (miR-31) expression is associated with the pathogenesis of psoriasis. The microarray determination of miR-31 expression and quantitative detection of the miR-31 target gene expression showed that the expression of RNF144B in the psoriasis group was higher than that of the control group. It was further revealed that RNF144B promotes T-lymphocyte activation by inhibiting the proliferation of dermal mesenchymal stem cells (DMSCs) and thereby participates in the pathogenesis of psoriasis [60].

## 9 RNF144 family proteins with other diseases

Prognostic indicators of head and neck squamous cell carcinoma (HNSCC) were screened using molecular characterization databases, the TCGA dataset, and the GEO dataset, and the results revealed that RNF144A has significant prognostic value in HNSCC patients and that RNF144A is involved in signaling pathways of protein metabolism and ubiquitination [61]. RNF144A is frequently mutated or epigenetically silenced in cancers, providing a theoretical basis for assessing the loss of function of RNF144A in tumorigenesis [62]. Exposure of mice to oncogenic conditions after the knockdown of RNF144A resulted in an increased incidence of bladder cancer [62]. This is attributed to the fact that RNF144A interacts with PD-L1 in the cytoplasmic membrane and intracellular vesicles and promotes polyubiquitination and degradation of PD-L1, while knockdown of RNF144A can stabilize PD-L1 [62]. Varicella-associated kinase 3 (VRK3) is an ERK negative regulator that inhibits ERK-dependent apoptosis as a means of exerting cytoprotective effects [63,64,65]. It was found that RNF144A can interact with VRK3 to promote the ubiquitination degradation of VRK3, activate ERK, and promote apoptosis, and these effects are intensified under conditions of oxidative stress [63].

When cells were subjected to sustained or severe DNA damage, the expression of RNF144A was elevated in a p53-dependent manner, and RNF144A was found to interact with the catalytic subunits of DNA-dependent protein kinases (DNA-PKcs) *in vivo* and *ex vivo* to promote their ubiquitinated degradation [66]. This finding suggests that RNF144A may be involved in the p53-mediated apoptosis by downregulating DNA-PKcs [66]. It was reported that the histone deacetylase inhibitor TMU-35435 induced RNF144A to interact with DNA-PKcs, and RNF144A promoted the ubiquitination of DNA-PKcs as a combined treatment to induce apoptosis and autophagic cell death [67]. Epidermal growth factor receptor (EGFR) belongs to the family of receptor tyrosine kinases (PTKs) [68]. Ligands of EGFR, such as EGF and the transforming growth factor α, can initiate EGFR dimerization, ubiquitination, activation, and endocytosis upon binding to other ligands, and activation of the EGF/EGFR signaling pathway was found to result in cell migration, proliferation, and differentiation [69,70]. RNF144A and EGFR were reported to interact with each other to promote the ubiquitination of EGFR, maintain EGFR protein stabilization, and prolong EGF/EGFR signaling. Knockdown of RNF144A reduced the EGF-dependent cell proliferation [71]. A specific AluYd8 progenitor exists in the intron of the RNF144A gene, and 14 transcribed sequences are initiated in the opposite direction of the RNF144A gene, i.e., antisense transcripts, whose transcriptional activation may interfere with the transcription of the RNF144A gene or block the binding of the transcripts in the promoter [72].

The most common genetic mutation in human cancers is the p53 gene mutation [73]. Isolation and functional analysis of target genes showed that RNF144B is associated with cell cycle control, which is related to the regulation of p21WAF1/CIP1 by RNF144B. As a direct transcription target of p53, the products of p21WAF1 can inhibit cell proliferation by binding directly to the subunits of cell cycle protein-dependent kinase or E2F transcription factors or by inhibiting DNA replication through interaction with the proliferating cell nuclear antigen. RNF144B interacts with and promotes the ubiquitinated degradation of p21WAF1, and HCT116 cells with RNF144B overexpression can lead to decreased p21WAF1 protein expression when exposed to γ-rays. Inhibition of the RNF144B expression by antisense oligonucleotides was reported to lead to accumulation of the p21WAF1 protein [74,75]. It was found that RNF144B induces p53-dependent apoptosis through its C-terminal TCD structural domain, but not through the RING-IBR-RING structural domain, suggesting that this process is not related to the ubiquitination function of RNF144B [10]. In addition, p53-46F, a mutant type of p53, induced higher levels of RNF144B expression than the wild-type p53 [76]. p73 is a p53-related transcription factor belonging to the p53 transcription factor family, and TAp73 and ΔNp73 are two isoforms of p73 (the former pro-apoptotic and the latter inhibiting apoptosis) [77,78,79]. It was reported that TAp73 can induce RNF144B, which then promotes the ubiquitinated degradation of ΔNp73, and this differential regulation maintains the stability of TAp73 and ΔNp73 [80]. This finding provides a therapeutic pathway to enhance the chemosensitivity of tumor cells. Apoptin, which is a protein derived from the chicken anemia virus, can induce cell death in various cancer cells. More specifically, Apoptin induces the expression of TAp73 to result in apoptosis [81]. It was also found that Apoptin induces the expression of RNF144B, leading to degradation of ΔNp73 and activation of the pro-apoptotic target PUMA, which eventually results in cancer cell death [82].

## 10 Conclusion

RNF144A and RNF144B are “ancient” genes, first reported in 1982 [83], while they are also “time-honored” genes, which are now reported every year [24,46]. Their involvement in organ pathologies in almost every system (Table 1) emphasizes their importance in human beings.

**Table 1:** Role and mechanism of RNF144A/RNF144B in different diseases

Diseases		RNF144A/RNF144B	Mechanisms	Results
Neuropsychiatric system	Glioblastoma	RNF144A	Binds directly to BMI1 and promotes its ubiquitination degradation	Negative correlation with patient survival
	Gliomas	RNF144A	ZDHHC18 and ZDHHC23 Competitively interacts with RNF144A to regulate polyubiquitination of BMI1	Negative correlation with patient survival
	Schizophrenia	RNF144A	Influences on antipsychotic drug side effects	Triglyceride disorders
	Chordoma	RNF144B	Promotes tumor cell proliferation, colony formation, invasion, migration, epithelial-epithelial mesenchymal transition and glycolysis	Promoting the progression of chordoma
Respiratory system	LUAD	RNF144A	Inhibits ALK-RNF144A fusion gene	Prolonged patient survival
		RNF144B	Relates to NADH dehydrogenase complex assembly, ubiquitin protein transferase activity, cytoplasmic DNA sensing pathway, RIG-I like receptor pathway, and NF-κB pathway	Significantly associated with clinicopathologic parameters and prognosis in LUAD
		RNF144B	Mediates cell cycle progression, DNA damage response and protein degradation	Suppression of LUAD
Digestive systems	HCC	RNF144A	Promotes ubiquitination of DNA-PKcs, inhibits TOP1, and enhances the radio-anti-HCC effect of NK cell activation	Increasing sensitivity of radiation therapy for HCC
	SAP	RNF144A	May modulate the permeability characteristics of immune cells in the microenvironment	Predicting the prognosis of SAP
	Gastric cancer	RNF144A	Promotes M2 macrophage migration	Oncogenic
		RNF144B	Mediates ubiquitination degradation of pirh2 and promotes p53 protein degradation	Relevant
Reproductive system	Epithelioid ovarian carcinoma	RNF144A	Reduces sphere formation and down-regulation of stem cell markers in EOC cells	Relevant
	Breast cancers	RNF144A	Interacts with YY1 and promotes its ubiquitination degradation, thereby inhibiting GMGF expression	Inhibition of proliferation, migration and invasion of breast cancer cells
			Interacts with HSPA2 and promotes its ubiquitination degradation	Suppressing breast cancers
	PCOS	RNF144B	Unknown	Relevant
	Ovarian cancer	RNF144B	Interacts with p21 and regulates degradation of the p21/p53 complex to promote Bcl-2 expression	Promoting growth and metastasis of ovarian cancer
	Endometriosis	RNF144B	Unknown	Relevant
	Endometrial cancer	RNF144B	Stabilizes the phosphorylation level of signaling molecules downstream of GSK3β	Promoting cell proliferation
	Spermatogonial stem cell proliferation	RNF144B	Interacts with FCER2 and downregulates the expression of the NOTCH2 structural domain N2ICD	Promoting the proliferation of human spermatogonial stem cells
Hematologic system	Multiple myeloma	RNF144A	Possibly relates to PVT1	Relevant
	Leukemia	RNF144B	Promotes ubiquitination degradation of NPMs	Relevant
	Lymphomas	RNF144B	Possibly regulates cell cycle progression	Relevant
Infectious diseases	Antiviral immunity	RNF144A	Interacts with STING and promotes ubiquitination of its K6 linkage at K236, thereby inducing downstream signaling molecules	DNA viruses play a protective role in inducing viral immunity
	Atlantic salmon infected with salmonella.	RNF144A	Strong induction by Salmonella and correlation with T cell function	Relevant
	Sepsis	RNF144B	Interacts with TBK1 to inhibit NF-κB activation and reduce inflammatory factor release	Reducing the inflammatory response due to sepsis
	LPS-induced inflammation	RNF144B	Interacts with TBK1 and inhibits TBK1 phosphorylation and K63-linked ubiquitination; inactivation of TBK1, dephosphorylation of IRF3, and reduced INF-β release	Inhibiting LPS-induced inflammatory response
			Unknown	Promoting the LPS-induced expression of IL-1β
	Poly(I:C)-induced inflammation	RNF144B	Knockdown of RNF144B leads to upregulation of poly(I:C)-induced expression of the transcription factor IRF3 as well as inflammatory factors such as TNFα, IL-6, and IFN-I	Negative regulation of poly(I:C)-induced inflammation
	Bovine tuberculosis	RNF144B	Unknown	Relevant
Dermatologic system	Keratinogenesis	RNF144B	Binds and mediates proteasomal degradation of ΔNp63α	Stabilizing proliferation and differentiation of keratinocytes
	Psoriasis	RNF144B	Inhibits proliferation of DMSCs to promote T lymphocyte activation	Relevant
Head and neck diseases	Squamous cell carcinoma	RNF144A	Participates in signaling pathways involved in protein metabolism and ubiquitination	Relevant
Urinary system	Bladder cancer	RNF144A	Interacts with PD-L1 and promotes polyubiquitination and degradation of PD-L1	Suppressing cancer

**Figure 8:**
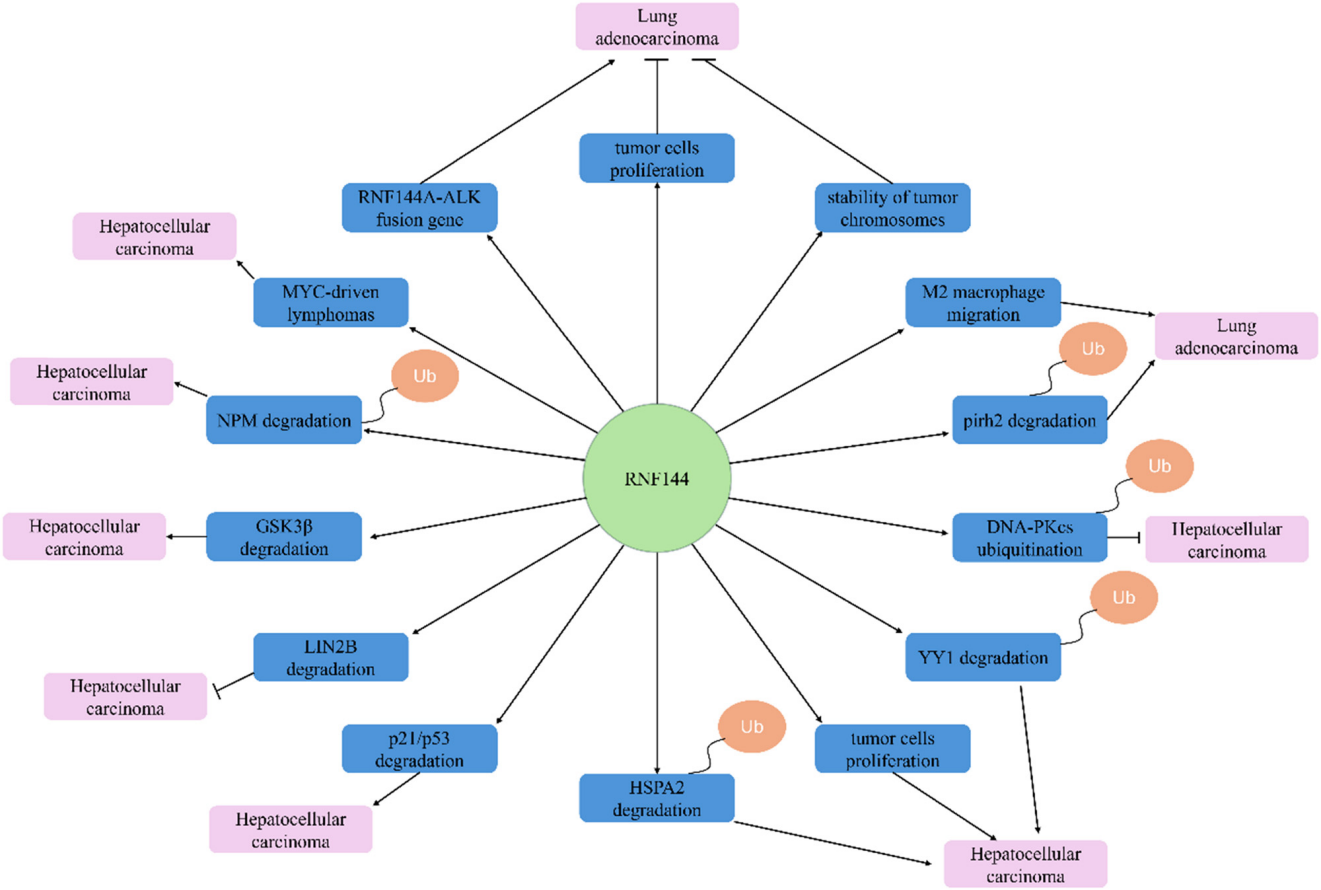
The dual roles of RNF144A/RNF144B in tumors.

By collating the literature on RNF144-related literature, we found that it is closely related to tumors. More noteworthy is the bidirectional role of RNF144 in tumors (Table 2, Figure 8). The tumor-promoting conditions and mechanisms are as follows: Formation of the RNF144A-ALK complex leads to RNF144A-promoted LUAD [22]. RNF144A promotes migration of M2 macrophages [25], and RNF144B promotes ubiquitinated degradation of pirh2 [26], which leads to RNF144 promoting gastric cancer. RNF144B promotes p21/p53 degradation in ovarian cancer [32]. RNF144B promotes phosphorylation of GSK3β in endometrial cancer [34]. RNF144B promotes ubiquitination degradation of NPM, which promotes leukemia [49]. RNF144B promotes MYC-driven lymphoma [50]. The tumor suppression and mechanism are as follows: RNF144B promotes the proliferation of tumor cells and also promotes the stabilization of tumor chromosomes, which leads to the suppression of LUAD by RNF144B [24]. RNF144A promotes the ubiquitination of DNA-PKcs and thereby inhibits HCC [29]. RNA144A promotes the degradation of LIN28B and thereby inhibits EOC [37]. RNF144A promotes ubiquitination degradation of YY1 [43], inhibits tumor cell proliferation and migration [44], and promotes ubiquitination degradation of HSPA2 [45], and these mechanisms lead to the inhibition of breast cancer by RNF144A.

**Table 2:** Dual role of RNF144 in tumors

Promote cancers	Inhibit cancers
**LUAD:**	**LUAD:**
RNF144A-ALK complex formation	RNF144B promotes tumor cell proliferation
**Gastric cancer:**	RNF144B promotes tumor chromosome stability
RNF144A promotes M2 macrophage migration	**HCC:**
RNF144B promotes pirh2 ubiquitination degradation	RNF144A promotes DNA-PKcs ubiquitination
**Ovarian cancer:**	**Ovarian cancer:**
RNF144B Promoting p21/p53 degradation	RNF144A promotes the degradation of LIN2B
**Endometrial cancer:**	**Breast cancer:**
RNF144B promotes ubiquitinated degradation of GSK3β	RNF144A promotes ubiquitinated degradation of YY1
**Leukemia:**	RNF144A inhibits tumor cell proliferation and migration
RNF144B promotes NPM ubiquitination degradation	RNF144A promotes ubiquitination degradation of HSPA2
**Lymphomas:**	
RNF144B promotes the driving role of MYC	

As a result, RNF144 promotes LUAD, gastric cancer, ovarian cancer, endometrial cancer, leukemia, and lymphoma and inhibits LUAD, HCC, ovarian cancer, and breast cancer. In addition, RNF144 is sometimes pro-carcinogenic and sometimes oncogenic in LUAD and ovarian cancer [22,23,24]. The mechanism underlying this phenomenon has been investigated to some extent, including gastric cancer, HCC, breast cancer, and leukemia, which are regulated by RNF144-mediated ubiquitination, and the degradation of p53 and LIN28B in ovarian cancer, which is also supposed to be RNF144-mediated ubiquitination, which needs to be further verified.

In addition to being associated with tumors, RNF144A and RNF144B play important roles in neurological and infectious diseases. Most of the literature demonstrates that RNF144 has an anti-infective effect. When the organism is infected by viral DNA or Salmonella, RNF144 promotes STING activation or modulation of T-cell function and production of interferon, which enhances antiviral immunity [51]. When cells were stimulated by poly(I:C), RNF144 downregulated the release of pro-inflammatory cytokines by an unknown molecular mechanism [56]. Interestingly, RNF144 also had a bidirectional regulatory effect on cells after monocyte-macrophage induction by LPS. Zhang et al. demonstrated that after LPS induction in macrophages, RNF144 promoted ubiquitinated degradation of TBK1, leading to a decrease in interferon release and thus anti-inflammation [54]. Paradoxically, Ariffin et al. found that the release of IL-1β was reduced after LPS-induced RNF144B knockdown macrophages, while it increased after LPS-induced RNF144B overexpressing macrophages and thus pro-inflammatory [55]. This finding has not been verified in great detail, and its mechanism still needs to be further investigated.

In neuropsychiatric disorders, RNF144 has consistent negative effects, promoting tumors, including gliomas and chordomas, or causing side effects from antipsychotics [15,18,19,20,21]. Considering the bidirectional regulatory role of RNF144 in other tumors and infectious diseases, it may have a similar role in other neurological disorders, and we can further investigate in this direction.

In addition, we found that RNF144A and RNF144B, although both members of the RNF144 family, do not have identical regulatory roles in relation to disease pathways. In some diseases, they have opposite regulatory roles. For example, in LUAD, RNF144A promotes tumors while RNF144B inhibits them [22,23,24]. In certain diseases, they have coordinated and complementary roles. For example, in gastric cancer, RNF144A and RNF144B jointly promote tumor development by promoting M2 macrophage migration and ubiquitination degradation of pirh2, respectively [25,26]. This should be related to the difference in the number of amino acids or the different TM structural domains at the C-terminus.

Unlike the bidirectional role of RNF144, other RBR E3s usually have a concerted role. For example, parkin, which has been shown to have a promoting effect in tumors such as HCC [84,85], drives apoptosis in HCC cells by inhibiting the NF-κB pathway by promoting the degradation of TRAF2 and TRAF6 [84,86]. HOIP is considered a protective factor for the organism. When HOIP is defective in the organism, it is more likely to acquire immunodeficiency [87]. This is because HOIP increases the ubiquitination of STAT1, which inhibits interferon production [88].

As ubiquitination-associated genes, RNF144A and RNF144B mostly regulate the activity of target proteins and signaling pathways by interacting with these proteins to promote their ubiquitination, mainly by facilitating the assembly of the K6-, K11-, K48-, and K63-linked ubiquitin chains [89]. However, the specific ubiquitination mechanisms of RNF144A and RNF144B in different diseases are currently understudied. There is only one adequate study reporting that RNF144A interacts with STING and promotes the ubiquitination of its K6 linkage at K236, which in turn induces downstream signaling molecules and protects against DNA viruses in inducing viral immunity [51]. Therefore, the mechanism underlying the ubiquitination modification of RNF144A still needs to be further explored, which also provides more research possibilities for developing novel disease-targeted therapies.

Most of the experimental results in the current literature on RNF144 are based on *in vitro* models, with relatively few *in vivo* studies, so the conclusions available may be limited. More mice and human specimens will be needed in the future to verify the reliability of these experimental results.

For now, RNF144 is involved in regulatory roles in numerous diseases, and exploring a criterion as a biomarker for diagnosing diseases, especially digestive and reproductive tumors, is worthy of our consideration, and the development of targeted biologics specific for RNF144 is a new direction for targeted therapy. However, as we mentioned earlier, RNF144 has bidirectional roles in tumors and inflammation, which makes it challenging to identify RNF144 as a criterion for disease diagnosis, and there is a long way to go to investigate new drugs targeting RNF144. We need to study the molecular mechanism of RNF144 regulation of diseases more profoundly to provide a reliable theoretical basis for diagnosis and targeted therapy.
